# Long-Term Outcome after Laparoscopic Bowel Resections for Deep Infiltrating Endometriosis: A Single-Center Experience after 900 Cases

**DOI:** 10.1155/2014/463058

**Published:** 2014-04-29

**Authors:** Giacomo Ruffo, Filippo Scopelliti, Alberto Manzoni, Alberto Sartori, Roberto Rossini, Marcello Ceccaroni, Luca Minelli, Stefano Crippa, Stefano Partelli, Massimo Falconi

**Affiliations:** ^1^Department of General Surgery, Sacro Cuore Don Calabria General Hospital, Via Don A. Sempreboni 5, 37024 Negrar, Italy; ^2^Department of Obstetrics and Gynecology, Sacro Cuore Don Calabria General Hospital, Via Don A. Sempreboni 5, 37024 Negrar, Italy

## Abstract

*Background*. Laparoscopic bowel resections for endometriosis are safe and effective but only short-term follow-up has been evaluated. In the present study long-term outcome in terms of intestinal and urinary function, fertility, chronic pain, and recurrence was assessed. *Materials and Methods*. From January 2002 to December 2010 nine hundred patients underwent laparoscopic bowel resection for endometriosis, and on 774 (86%) a questionnaire was administered. Patients were divided into 3 groups on the strength of the operation date. Postoperative diarrhea, constipation, rectal bleeding, tenesmus, dyschezia, dysuria, dyspareunia, fertility, and recurrence of disease were assessed. *Results*. The median follow-up was 54 months (range 1–120). All the evaluated symptoms significantly improved over time, with *P* = 0.0001 for dyspareunia, constipation, and pelvic pain and *P* = 0.004 for diarrhea. Nonsignificant improvement was reported for dysuria and rectal bleeding (with *P* = 0.452 and *P* = 0.097, resp.). *Conclusions*. The present results confirm that bowel resections for endometriosis are correlated with an acceptable complication rate even at long-term follow-up and that symptoms significantly improve over time, except for rectal bleeding and dysuria, the latter associated with a neurological damage.

## 1. Introduction

Endometriosis is a benign disease but it can seriously worsen quality of life. Its incidence is quite high, affecting 6–10% of women in childbearing age [1, 2]. Deep infiltrating endometriosis (DIE) is a form of endometriosis in which the pathologic tissue can penetrate up to 5 mm under the surface of the affected structure [1, 2]. The incidence of DIE is reported in 20% of all cases of endometriosis and the gastrointestinal tract results involved in 5.3–12% [3, 4]. The most frequent localization is the rectosigmoid junction and it has been estimated in 65% of cases; other common localizations are the ileocaecal junction in 20% and the rectum in 15%. The endometriotic tissue can involve the submucosal layer but the infiltration of the mucosa is very rare.

Bowel endometriosis may cause severe symptoms such as diarrhea, constipation, abdominal pain, bleeding, dyschezia, and rarely bowel obstruction. The best therapeutic approach is still controversial but several studies have demonstrated that surgery offers improvement of symptoms, better quality of life, and acceptable postoperative fertility rates [5–7]. For this purpose it is essential to establish a long-term outcome to evaluate intestinal and urinary dysfunctions, quality of life, and fertility rate [8, 9].

Recently, a nerve-sparing approach laparoscopic bowel resection for DIE was proposed to preserve bladder, rectal, and sexual functions [10, 11].

Since Nezhat described in 2001 the first laparoscopic bowel resection for endometriosis [12], many studies have been published on this topic and recently, Daraï et al. have demonstrated, in a prospective trial, that laparoscopy is a safe option in the treatment of bowel endometriosis and offers a high pregnancy rate and a good quality of life [13].

In our division, since January 2002, we performed 1023 colorectal resections for bowel endometriosis; after 10 years, on the basis of this experience, we decided to analyse retrospectively our results. The aim of this study was to investigate bowel and urinary dysfunction and fertility rate in a large series of bowel laparoscopic resection for DIE.

## 2. Materials and Methods

Between January 2002 and December 2010, 1023 women underwent laparoscopic bowel resections for DIE at the Departments of General Surgery and Gynecology, Ospedale “Sacro Cuore-Don Calabria,” Negrar, Verona. Discoid and transvaginal resections were excluded from the present study and 900 patients were considered eligible for the investigation.

All patients were postoperatively interviewed by telephone between January 2011 and March 2011 by three male surgeons, and a questionnaire was filled in forms for each patient, dividing the whole population into three subgroups of 300 patients; the first group included patients treated between 1 January 2002 and 31 March 2006, the second from 1 April 2006 to 1 February 2009, and the third up to 31 December 2010.

**Figure 1:**
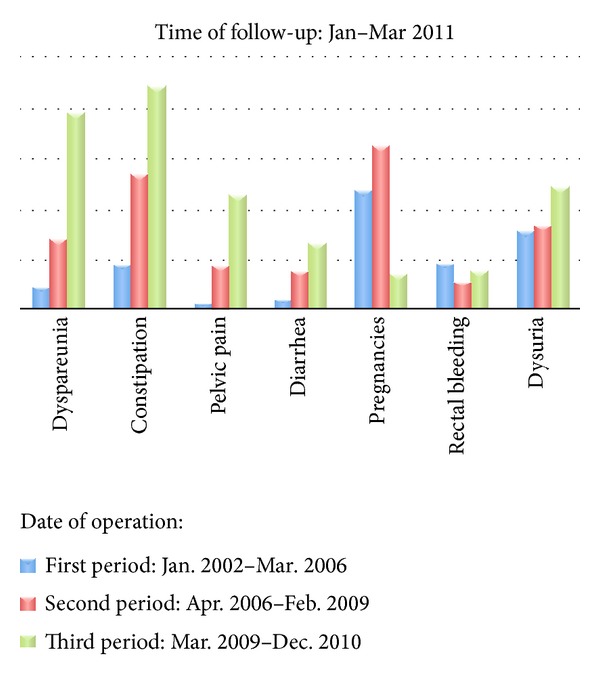


The questionnaire is made up of questions regarding intestinal and gynaecological symptoms: presence of diarrhea or constipation, rectal bleeding, postoperative pregnancy, dyspareunia, dysuria, and pelvic or back pain, evaluated using a 10-point visual analogue scale (0 = absent, 10 = unbearable) (Figure 1).

All women underwent a clinical multidisciplinary evaluation; they were all studied with a barium enema and a transvaginal ultrasound. The indication for surgery was made on the basis of the presence of intestinal stenosis associated with intestinal-related symptoms. All women underwent laparoscopic bowel resection after a complete multidisciplinary evaluation. The procedures were carried out when a stenosis of the intestinal lumen was radiologically showed, when gastrointestinal symptoms were described, and when the presence of DIE was intraoperatively demonstrated. Ureteroneocystostomy was performed in patients with radiological stenosis of the ureter and hydroureteronephrosis. All patients had a stage IV endometriosis according to the American Society of Reproductive Medicine AFs, 1989, and all of them underwent a bowel resection (rectal, rectosigmoid, sigmoid, ileocaecal, or ileal resection). For rectal, rectosigmoid, or sigmoid resections, an end-to-end anastomosis was performed, according to the Knight-Griffen procedure, with manual or mechanical sutures [10]. Resections were classified according to distance from the anus as high/medium (>8 cm), low (5–8 cm), or ultralow (<5 cm) and measured by intraoperative proctoscopy. Ileocaecal and ileal resection were carried out extracorporeally with a manual anastomosis through, either a sovrapubic or a left or right pararectal incision. All patients were operated on by the same surgical, gynaecological, and urological teams with surgical standardized techniques. We retrospectively analyzed diarrhea, constipation, rectal bleeding, tenesmus, dyschezia, dysuria, fertility after surgery, and relapse. Constipation was defined as fewer when three difficult, incomplete, or infrequent evacuations of dry hardened faeces per week, even with laxatives or enemas, appeared after surgery. Preoperative and postoperative scoring of symptoms was considered using a 10-point visual analogue scale (VAS; 10-point rating scale: 0 = absent, 10 = unbearable) [14]. All the data obtained by the questionnaire were investigated and inserted in a computerized database.

One-way ANOVA was performed to compare all variables in the two study groups. Categorical variables were compared by using *χ* ^2^ test. Wilcoxon Signed Rank test was adopted to compare paired scale variables. Statistical analyses were performed using SPSS for Windows 16.0 package (SPSS Inc., © Copyright IBM Corporation 2010 IBM Corporation, Route 100 Somers, NY).

## 3. Results

After a median follow-up of 54 months (range 1–120), 900 women were called by telephone; long-term follow-up data were available for 774 patients (86%), 210 for the first period (70%), 267 for the second (85%), and 297 for the third (99%). No significant differences in demographic data between the three groups were reported (Table 1). Median age was 27.5 (range 22–51) and median BMI (body mass index) was 23.7 (range 18.5–31.5). All women were at fertile age and 123 (15.8%) of them reported a previous pregnancy before surgery. Five hundred and eighty-three (64.8%) patients had previous abdominal surgery, laparotomy in 252 cases and laparoscopy in 331. The data related to the operation are reported in Table 2. Among the whole population (900 women), sixty-three percent of patients (567) underwent a rectosigmoid resection, 25% (225 patients) underwent a rectal resection, 5% (45 patients) underwent an ileocaecal resection, 61 patients (6.8%) underwent a double resection (rectosigmoid and ileocaecal), and 2 patients (0.2%) underwent a triple resection (rectosigmoid, ileal, and ileocaecal). One hundred and fifty-six (21.3%) patients required an ileostomy. In 5 cases the ileostomy was still present at the time of follow-up. In 60 cases (7.7%) a ureteral reimplantation was performed. The results were divided per period and reported in Table 3. After the operation, we registered 128 (16.5%) pregnancies, among which 35 (5.3%) were in the nulliparous group that consisted of 651 women; twenty-four patients (3.5%) reported at least one miscarriage. Forty-eight pregnancies were reported in the first period, 65 in the second, and 15 in the third. One hundred and sixteen patients (14.9%) reported dyspareunia (median VAS 6, range 1–10), 9 in the first period (4.2%), 29 in the second (10.8%), and 78 in the third (26.2%). Ninety-five percent of patients reported tenesmus in the first month following surgery but all of them described this symptom as completely settled at the time of follow-up. One hundred and sixty-two patients (21%) reported constipation, 18 in the first period (8.5%), 54 in the second (20.2%), and 90 in the third (30.3%); all of them underwent a rectosigmoid resection (157 anterior rectal resections with low anastomosis in 143 cases and ultralow in 14) except in 5 cases, 2 ileocolic resections and 3 combined ileocolic and rectal resections. Fifty-seven (7.3%) patients reported diarrhea; 55 of them had a rectosigmoid resection and two an ileocaecal resection. This symptom was present in 4 cases (1.9%) in the first period, in 16 cases (5.9%) in the second, and in 27 cases (9.1%) in the third. Forty-seven (6.0%) women reported mild rectal bleeding: one of them had an ileocaecal resection and 46 had a rectal resection. This symptom was present in 19 cases (9.0%) in the first period, in 12 cases (4.5%) in the second, and in 16 cases (5.3%) in the third. Sixty-four (8%) women reported pelvic pain, 3 in the first period (1.4%), 18 in the second (6.7%), and 46 in the third (15.4%); median VAS was 6 (range 1–10). One hundred and fourteen (14.7%) women described dysuria, 31 in the first period (14.7%), 34 in the second (12.7%), and 49 in the third (16.4%); median VAS was 5.5 (range 1–10). Among the 60 patients who underwent a ureteral reimplantation, only 2 of them complained of this symptom. Twenty-three women (2.9%) reported a relapse; in 16 cases (2%) it involved the gynaecological apparatus, in 5 cases (0.6%) the urinary system, and in 2 cases (0.2%) the intestinal tract. Five of them (0.5%) were reoperated on but no bowel resection was necessary. At univariate analysis for the different periods, symptoms as dyspareunia, constipation, and pelvic pain statistically improved over time with *P* = 0.0001, as well as diarrhea with *P* = 0.004. As regards dysuria and rectal bleeding, no improvement was reported over time, with *P* = 0.452 and *P* = 0.097, respectively.

## 4. Discussion and Conclusion

Several studies demonstrate that colorectal resection for DIE is a safe and effective procedure with an acceptable postoperative complication rate and that it improves significantly quality of life; however, only short-term postoperative outcome has been evaluated [2, 4].

Many records analysed functional results after sphincter preserving colorectal resection, low anterior rectal resection, and abdominoperineal resection in treatment of rectal cancer [15]. In case of surgery for malignant disease the bowel function is related to the length of bowel resected, to preoperative chemoradiotherapy, and to the surgical technique applied as demonstrated by several studies [16]. Bowel function disorders, such as constipation and diarrhea, after colorectal resection for cancer are very common [17]. In case of colorectal resection for DIE the bowel dysfunction rate is significantly lower, probably because of the young age of the patients, because radiochemotherapy is not necessary, and because, in the majority of cases, the length of the resected colorectal segment is shorter and limited to the affected tract; in fact oncological radicality is not necessary.

Bowel movements disorders are difficult to evaluate since the latter depend on several factors among which the surgical technique can be considered the most important. Postoperative constipation can be correlated to the hypertension of the puborectalis sling, which can be frequently investigated by a radiological defecography. At our institution, patients who suffer from this type of dysfunction undergo a physiatric evaluation and a biofeedback pelvic floor therapy, which proved to be very useful for many women. Very few patients complained of rectorrhagia in our series; among them no cases of recurrence were recorded. Probably this symptom is correlated to a concomitant proctological disease; in fact its incidence in the three periods is homogeneous and it is not influenced by the length of the follow-up.

The urinary disorders after colorectal resection for endometriosis worsen significantly quality of life and the incidence is reported between 0.5 and 19.5% [8, 9]. In our series patients who suffered from dysuria were 114 (14.7%) and its rate is homogeneous in the three periods. Many studies describe urinary dysfunctions after intestinal resection for endometriosis as for laparoscopic surgery for malignant diseases; they are most probably correlated to inferior hypogastric plexus damage; in fact this symptom in our series did not improve over time after the operation, according to the hypothesis that the complication is correlated to a neurological impairment. Different techniques have been described in gynecological surgery to preserve these neural fibers such as the Tokyo method [18]; however very few long-term results are available. Recently, Ceccaroni et al. reported, in a single-center prospective study performed on 126 patients, good results of the nerve-sparing radical excision of DIE with segmental bowel resection in terms of reduced bladder dysfunction and higher satisfaction than the classical technique [10].

Postoperative recurrence rate in our series is very low even at long-term follow-up. If second surgery was needed for persistence or recurrence of the disease, no bowel resection was necessary to achieve a complete ablation of endometriosis.

Postoperative pregnancy rate is reported in literature between 45 and 48% [5]. In our series the rate is lower but none of our patients underwent a preoperative fertility study, and the resort to assisted conception was not investigated. Some studies demonstrated that fertility is influenced by colorectal surgery even for nongynecologic diseases; for example, Gorgun et al. reported a statistically significant difference in fertility before and after surgery in patients who underwent coloproctectomy for hemorrhagic enterocolitis [19]. Fedele et al. described how the pregnancy rate diminishes in case of recurrence of endometriosis, and actually the five patients in our study who had a reoperation for recurrence did not become pregnant [20]. Some authors described how postoperative complications could reduce the possibility of pregnancy and they advise an ovariopexy to keep the ovaries away from the affected areas [21, 22]. In the present study the correlation between postoperative complications and pregnancy rate was not investigated; therefore this issue merits further studies.

As regards complications as dyspareunia, constipation, diarrhea, and pelvic pain, these symptoms significantly improve over time, rising at a very low rate at long-term follow-up.

In conclusion the present study confirms that bowel resections for endometriosis have an acceptable postoperative complication rate even at long-term follow-up and symptoms improve over time, although our data concern a very wide range of follow-up time, which can be considered as a bias. For this reason we believe that further studies are needed to confirm our results.

## Figures and Tables

**Table 1:** Demographic data.

Age (years)	27.5 (22–51)
BMI (Kg)	23.7 (18.5–31.5)
Previous pregnancies	123 (15.8%)
Previous abdominal surgery	583 (64.8%)

**Table 2:** Types of operations.

Rectosigmoid	567 (63%)
Rectal	225 (25%)
Ileocecal	45 (5%)
Rectosigmoid + ileocecal	61 (6.8%)
Rectosigmoid + ileocecal + ileal	2 (0.2%)
Ileostomy	156 (21.3%)
Ureteral reimplantation	60 (7.7%)

**Table 3:** Results.

	Total 774 patients	First period January 2002–March 2006 (*n* = 210)	Second period April 2006–February 2009 (*n* = 267)	Third period March 2009–December 2010 (*n* = 297)	*P* value	
Pregnancies	128 (16.5%)	48 (22.8%)	65 (24.3%)	15 (5.0%)	*P* < 0.0001	S
Dyspareunia	116 (14.9%)	9 (4.2%)	29 (10.8%)	78 (26.2%)	*P* < 0.0001	S
Constipation	162 (21%)	18 (8.5%)	54 (20.2%)	90 (30.3%)	*P* < 0.0001	S
Diarrhoea	57 (7.3%)	4 (1.9%)	16 (5.9%)	27 (9.1%)	*P* = 0.004	S
Rectal bleeding	47 (6.0%)	19 (9.0%)	12 (4.5%)	16 (5.3%)	*P* = 0.097	NS
Pelvic pain	64 (8%)	3 (1.4%)	18 (6.7%)	46 (15.4%)	*P* < 0.0001	S
Dysuria	114 (14.7%)	31 (14.7%)	34 (12.7%)	49 (16.4%)	*P* = 0.452	NS
